# *Boswellia serrata* Resin Extract in Diets of Nile Tilapia, *Oreochromis niloticus*: Effects on the Growth, Health, Immune Response, and Disease Resistance to *Staphylococcus aureus*

**DOI:** 10.3390/ani11020446

**Published:** 2021-02-08

**Authors:** Metwally M. Montaser, Mohamed E. El-sharnouby, Gamal EL-Noubi, Heba M. El-Shaer, Alshimaa A. Khalil, Mohamed Hassanin, Shimaa A. Amer, Doaa A. El-Araby

**Affiliations:** 1 Department of Science and Technology, University College, Ranyah, Taif University, P.O. Box 11099, Taif 21944, Saudi Arabia; m.montaser@tu.edu.sa; 2 Department of Biotechnology, College of Science, Taif University, P.O. Box 11099, Taif 21944, Saudi Arabia; m.sharnouby@tu.edu.sa; 3 Department of Fish Diseases and Management, Faculty of Veterinary Medicine, Zagazig University, Zagazig 44511, Egypt; elnobidoc@hotmail.com (G.E.-N.); hebaelshaer22@gmail.com (H.M.E.-S.); shvet2013@gmail.com (A.A.K.); hassanin1974@yahoo.com (M.H.); 4 Department of Nutrition and Clinical Nutrition, Faculty of Veterinary Medicine, Zagazig University, Zagazig 44511, Egypt; 5 Department of Fish Health and Management, Central Laboratory for Aquaculture Research (CLAR), Agriculture Research Center, Abbassa, Abo-Hammad, Sharkia 44662, Egypt; dr.doaae@gmail.com

**Keywords:** *Boswellia serrate* resin extract, *Oreochromis niloticus*, growth, histoarchitecure, immunity, *Staphylococcus aureus*

## Abstract

**Simple Summary:**

The current study evaluated the effects of *Boswellia serrata* resin extract (BSRE) as a feed additive on the growth performance, immune response, antioxidant status, and disease resistance of Nile tilapia, *Oreochromis niloticus*. Fish were fed on four basal diets complemented with four levels of BSRE 0, 5, 10, or 15 g kg^−1^. The results of this study proposed that BSRE addition can enhance the antioxidant activity, immune status, and disease resistance of *O. niloticus* to *S. aureus* infection. The level of 5 g kg^−1^ BSRE can improve fish growth without causing harmful effects on fish health. Higher levels of BSRE are not recommended as they badly affected the histoarchitecture of many vital organs.

**Abstract:**

The influences of *Boswellia serrata* resin extract (BSRE) as a feed additive on the growth performance, immune response, antioxidant status, and disease resistance of Nile tilapia, *Oreochromis niloticus* L. were assessed. One hundred-forty four fingerlings (initial weight: 21.82 ± 0.48 g) were randomly allotted into four groups with three replicates where they were fed on one of four treatments with four levels of *Boswellia serrata* resin extract 0, 5, 10, or 15 g kg^−1^, BSRE0, BSRE5, BSRE10, BSRE15, respectively for eight weeks. After the end of the feeding trial, the fish were challenged with *Staphylococcus aureus*, and mortalities were noted. The final body weight, total body weight gain, and the total feed intake were quadratically increased in BSRE5 treatment (*p* < 0.01). The protein productive efficiency (PPE) was linearly and quadratically increased in all BSRE supplemented treatments (*p* < 0.01). Dietary addition of BSRE raised the fish crude protein content and reduced the fat content in a level-dependent manner (*p* < 0.01). The ash content was raised in the BSRE15 group (*p* < 0.01). Dietary BSRE supplementation decreased the serum levels of glucose, total cholesterol, triglycerides, and nitric oxide. It increased the serum levels of total protein, albumin, total globulins, α1 globulin, α2 globulin, ß globulin, ɣ globulin, Catalase, and SOD (superoxide dismutase) activity, GSH (reduced glutathione), lysozyme activity, and MPO (myeloperoxidase) in a level-dependent manner (*p* < 0.05). The BSRE15 diet increased the serum level of ALT (alanine aminotransferase) and decreased creatinine serum level (*p* < 0.05). Dietary BSRE supplementation increased the relative percentage of survival % (RPS) of *S. aureus* challenged fish. The histoarchitecture of the gills and kidney was normal in the BSRE5 treatment and moderately changed in BSRE10 and BSRE15 treatments. The splenic lymphoid elements were more prevalent, and the melano-macrophage centers (MMC) were mild to somewhat activated in BSRE supplemented treatments. Dietary BSRE supplementation improved the intestinal histomorphology. It can be concluded that BSRE addition can enhance the antioxidant activity, immune status, and disease resistance of *O. niloticus* to *S. aureus* infection. The level of 5 g kg^−1^ BSRE can improve fish growth without causing harmful effects on fish health. The highest levels of BSRE are not recommended as they badly affected the histoarchitecture of many vital organs.

## 1. Introduction

Recently, aquaculture production has witnessed a boom to fill the needs of the fish shop. Fish is the main food in most developing countries, which leads to increased demand [1]. Worldwide production of tilapia (*Oreochromis* spp.) has been increasingly growing at a rate of 10% since 2001, making it one of the main and rapidly developing aquaculture species [2]. Nile tilapia (*Oreochromis niloticus*) is one of the most widely cultured species in many tropical countries. The fish’s response to stress conditions depends on the stress factor (crowding, hypoxia, temperature, presence of heavy metals, etc.) and the fish features such as fish species, age, or gender. Such stressful conditions can increase the spread of pathogenic bacteria and cause severe disease outbreaks [3,4].

*Staphylococcus aureus* is a dangerous pathogen isolated from society and healthcare centers worldwide. The importance of *S. aureus* is owing to the rapid occurrence of antibiotic resistance among most of its isolates and the secretion of virulence factors that donate to their invasiveness and ability to disease [5]. Its pathogenicity is mostly related to genetic characteristics that mediate virulence, immune evasion, invasive capacity, and antibiotic resistance [6]. It is the third most common reason for foodborne disease worldwide and is the most common factor in food poisoning outbreaks [7]. The prohibition on antibiotics as feed additives has hastened research and resulted in extensive studies on alternative feed additives in aquaculture diets. These alternatives, such as phytogenic feed additives, include herbs, resins, or spices that enrich the diet with many volatile substances and aromatic compounds. These supplements enhance health status, growth performance, and fish immunity [8,9,10].

*Boswellia serrata* resin, olibanum or frankincense, is obtained from the Burseraceae family [11,12], which grows in dry and arid regions Yemen and Oman, India, northeast Africa [13]. This old medicine is thought to have antiseptic, anti-inflammatory, antimicrobial, anxiolytic, and anti-cancer effects [14]. These medicinal effects are related to various aromatic compounds, such as the main active principle boswellic acid [15,16]. Moreover, *boswellia serrata* resin contains volatile oils which composed of sesquiterpenes and monoterpenes [17,18], diterpenes such as cembrenol (serratol), incensole, and incensole acetate [18,19], lipophilic pentacyclic triterpene acids of the oleanane (α-boswellic acids), ursane-(β-boswellic acids) and lupane-type (lupeolic acids), and an ether-insoluble fraction containing polysaccharides (arabinose, galactose, xylose) [20]. It is approved that *Boswellia serrata* resin is safe, and its use as a feed additive is allowed by the US Food and Drug Administration (USFDA) [21]. The medicinal abilities of *Boswellia serrata* have been established by several investigators and have been well acknowledged in the works [22,23,24,25]. There are no data about their aptness for use in aquaculture and their effects on fish growth, immunity, and health status. So, this study, for the first time, evaluated the potential impacts of using *Boswellia serrata* resin extract on the growth performance, immune response, disease resistance, and antioxidant status of *Oreochromis niloticus*.

## 2. Material and Methods

The current study was carried out in the central laboratory for aquaculture research (CLAR), Abo-Hammad, Alsharqia, Egypt. The study protocol was confirmed by the Ethics of Animal Use in Research Committee of Zagazig University (ZU-IACUC/2/F/190/2019). The experimental procedures were accomplished following the National Institute of Health general guidelines for the Care and Use of Laboratory Animals in scientific investigations.

### 2.1. Boswellia Serrata Resin Extraction

**Figure 1:**
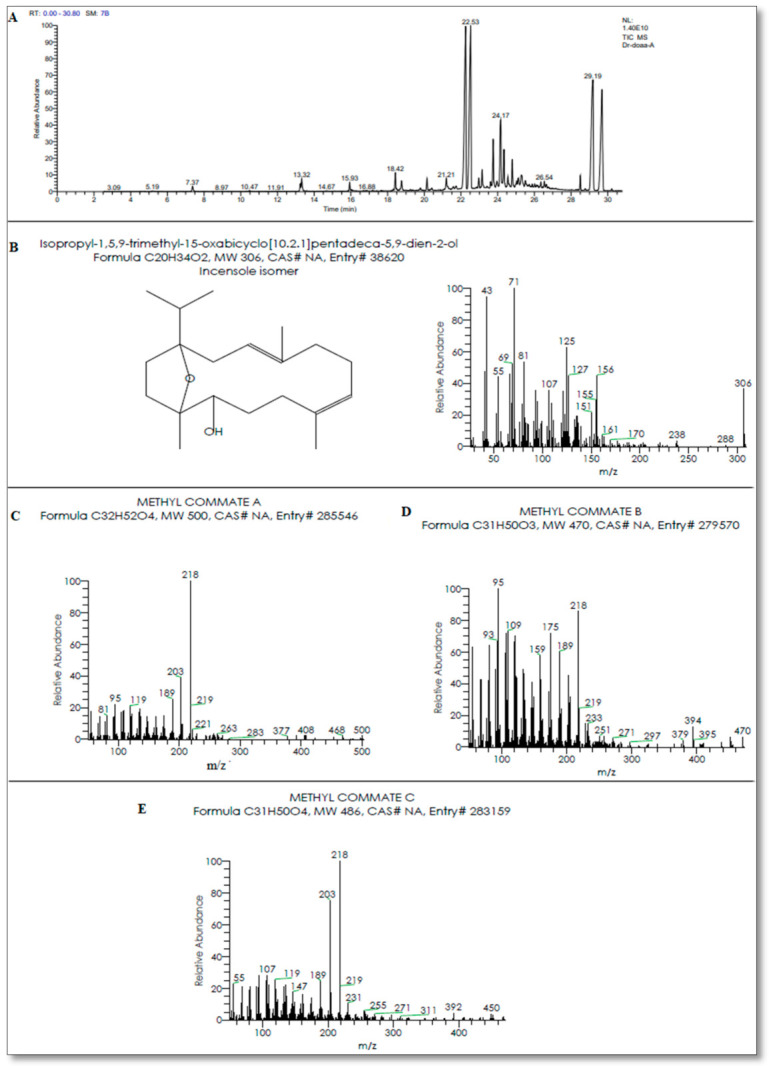
(**A**) Chromatographic characteristics by GC-mass techniques showing the active principles in *Boswellia serrata* resin extract (BSRE). (**B**) Diterpenes (Incensole, C_20_H_34_O_2_) with an area of 19.97%; (**C**) methyl Commate A (boswellic acids, C_32_H_52_O_4_); (**D**) methyl Commate B (Oleanolic acid, C31H50O3); (**E**) methyl Commate C (Sulfurenic acid, C31H50O4) with an area of 15.95, 15.95 and 13.63 %, respectively.

The hard, dry *Boswellia serrata* resin was grounded to a fine powder then extracted with ethyl alcohol (95%) by Soxhlet apparatus (VELP SCIENTIFICA) till complete extraction then evaporated to a thick syrup of brown color, which was undergoing Spectroscopic analysis (The GC-mass techniques, the regional center for Mycology and Biotechnology Al-Azhar University, Egypt) that revealed the richness of BSRE with terpenes (Figure 1A) especially diterpenes (Incensole isomer) with an area of 19.97% with the elemental analysis indicates the chemical formula is C_20_H_34_O_2_ (Figure 1B). The triterpenes, especially methyl Commate A (boswellic acids), methyl Commate B (Oleanolic acid), and methyl Commate C (Sulfurenic acid) with an area of 15.95, 15.95, and 13.63% for methyl Commate A, B, and C with the elemental analysis indicates the chemical formula as C_32_H_52_O_4_, C_31_H_50_O_3_, and C_31_H_50_O_4_ for methyl Commate A, B, and C, respectively (Figure 1C–E).

### 2.2. Fish and Cultural Conditions

Healthy *O. niloticus* fingerlings (N = 144, initial body weight 21.82 ± 0.48 g) were examined for pre-trial health status following the guidelines of the Canadian Council on Animal Care, CCAC [26] then randomly distributed in twelve 100-L static glass aquaria (12 fish/ aquarium) supplied with dechlorinated tap water with a daily exchange of about 25% of water and continuous aeration using electric air pumping compressors with an air stone for two weeks, and fish were fed on a basal diet before the onset of the experiment. The water quality parameters were kept under the same conditions, including temperature (28.3 ± 1.1 °C), dissolved oxygen (6.18 ± 0.4 mg/L), pH (6.9 ± 0.1), and ammonia (0.035 ± 0.01 mg/L) with a controlled photoperiod (12 h light: 12 h dark) in the laboratory according to American Public Health Association [27].

### 2.3. The Experimental Design and Diets Preparation

Fish were randomly allocated into four groups in triplicates (12 fish/replicate) for eight weeks. The experimental treatments consisted of basal diets complemented with four levels of *Boswellia serrata* resin extract 0, 5, 10, or 15 g kg^−1^, BSRE0, BSRE5, BSRE10, BSRE15, respectively. The basal diet (Table 1) was formulated and customized to fill the recommended nutritional needs of Nile tilapia, according to the National Research Council, NRC [28]. The ingredients were mechanically mixed and pelletized using a pellet machine, ultimately producing pellets of 1.5 mm diameter. The prepared diets were air-dried at room temperature for 24 h and stored in a refrigerator at 4 °C until use. Proximate chemical analysis of diets was carried out according to AOAC [29]. Fish were manually fed till satiation daily at 8.00 a.m. and 2.00 p.m.

### 2.4. Growth Performance, Proximate Chemical Composition of the Whole-Fish Body, and Economic Efficiency of the Feed

Fish were weighed at the beginning of the experiment, then fish body weights and feed intake were recorded every two weeks. The following growth performance parameters were calculated as follow;

Total body weight gain (TBWG) = Final body weight (g) − Initial body weight (g)

Average daily feed intake (ADFI) (g/fish/day) = total feed intake/ number of feeding days.

FCR = total feed intake (g)/total weight gain (g).

PER = total weight gain (g)/protein intake (g).

Protein productive value (PPV) = Protein gain (g)/protein intake (g).

The whole fish-body composition was analyzed at the beginning of the experiment (10 fish/stock) and the experiment’s end (three fish/ tank). Fish samples were analyzed for moisture, crude protein, crude fat, and total ash, according to AOAC [29].

Efficiency measures were calculated according to El-Telbany and Atallah [30], Dunning and Daniels [31] as follow:

Feed cost (USD) = the cost of one kg of each diet × the amount of total feed intake (kg) during the experimental period (70 days).

Feed cost/kg gain (USD) = Total feed cost/Total weight gain (kg).

### 2.5. Sampling

By the end of the feeding trial (8 weeks), nine fish/group were randomly selected and anesthetized with 95 mg L^−1^ clove oil (Oleum, Cairo, Egypt) within 3 min [32], and blood samples were collected from the caudal blood vessels of fish with clean and sterile syringes without anticoagulant then serum was separated by centrifuging at 3000 RPM for 10 min. The attained serum was used for the evaluation of some blood biochemical parameters. Moreover, gills, kidneys, spleen, and posterior intestinal tissues were sampled for further histological investigation.

### 2.6. Blood Biochemical Parameters

Total cholesterol, triglycerides, and glucose were measured by the colorimetric diagnostic kits of spectrum-bioscience (Egyptian Company for Biotechnology, Cairo, Egypt) following the procedures of Allain et al. [33], McGowan et al. [34], and Trinder [35], respectively. The qualitative fractionation of serum proteins was carried out using cellulose-acetate electrophoresis, according to Kaplan and Savory [36].

### 2.7. Antioxidant Activity

Superoxide dismutase (SOD) activity, catalase (CAT) activity, and reduced glutathione (GSH) level in fish serum were measured by commercial colorimetric kits purchased from Biodiagnostic Co., Cairo, Egypt, and following the procedures of Nishikimi et al. [37], Aebi [38], and Beutler [39], respectively.

### 2.8. Immunological Assessment

The lysozyme activity was analyzed using the lysoplate technique according to the method of Grinde [40]. The activity of MPO and NO level was measured following Quade and Roth [41] and Moshage [42], respectively.

### 2.9. Histopathological Investigation

Samples from the fish gills, kidney, spleen, and posterior intestinal tissues were collected and fixed in 10% neutral buffered formalin for 48 h, dehydrated in gradual ascending ethanol, cleared in xylene, and embedded in paraffin. The paraffin (5 micrometers thick) was sliced by a microtome (Leica RM 2155, Wetzlar, Germany). The sections were stained with hematoxylin and eosin [43]. Intestinal morphometric analysis was carried out according to Pirarat et al. [44].

### 2.10. Bacterial Challenge

After the feeding trial ended, all fish were intraperitoneally injected with the pathogenic bacterium *Staphylococcus aureus* at a dose of 0.2 mL suspension containing 4 × 10^6^ cells/mL by McFarland standard tubes. *Staphylococcus aureus* was isolated before from the dead fish and established to be pathogenic for *O. niloticus* by the Department of Fish Diseases and Management of the Faculty of Veterinary Medicine Zagazig University. *Staphylococcus aureus* identification was made by conservative biochemical tests and VITEK 2-C15 automated system (BioMérieux, Marcy-l’Étoile, France) following manufacturer’s instructions as described by [45,46] at Microbiology and Immunology Department, National Research Centre (NRC), Dokki, Giza, Egypt. All groups were kept under observation for 14 days to record any abnormal clinical signs and daily mortalities. Fish mortalities were used to calculate the relative percentage survival “RPS” according to the formula of Amend [47]. RPS = 100 − [(treatment mortality ÷ control mortality) × 100].

### 2.11. Statistical Analysis

Before statistical analysis, the normality of distribution and homogeneity of variances between different treatments were tested using the Kolmogorov–Smirnov test and Bartlett’s test, respectively, and the assumption was achieved (*p* > 0.05). ANOVA (Analysis of Variance) test was used based on polynomial orthogonal contrasts. Linear and quadratic regression equations were performed using SPSS (Statistical Package for Social Sciences) Version 17 for Windows (SPSS Inc., Chicago, IL, USA) at a significance value of *p* < 0.05. Post-hoc Tukey’s test was applied to determine differences among means, and the variation in the data was expressed as pooled SEM, and the significance level was set at *p* < 0.05.

## 3. Result

### 3.1. Growth Performance, Fish Whole-Body Composition, and Economic Value

The effects of dietary *Boswellia serrata* resin extract on the growth performance of *O. niloticus* are shown in Table 2. Quadratic increase in the FBW, TBWG, ADFI, and the total FI was observed in fish fed on the BSRE5 diet by 8.95%, 16.92%, and 15.15%, respectively, compared to those fed on the BSRE0 diet (*p* < 0.01). The protein productive efficiency was increased linearly and quadratically in all BSRE supplemented treatments by 45.71, 30.98, and 35.28% for BSRE5, BSRE10, and BSRE15, respectively (*p* < 0.01). The FCR and PER were not significantly affected by BSRE supplementation (*p* > 0.05). Based on the average daily feed intake, the daily dose of BSRE extract consumed per fish was 4.28 ± 0.07, 8.08 ± 0.27, 11.37 ± 0.18 mg for BSRE5, BSRE10, and BSRE15, respectively.

Table 3 shows the fish’s whole-body composition. The crude protein content was linearly and quadratically increased in BSRE supplemented diets by 44.62, 39.71, and 35.71% for BSRE5 BSRE10, and BSRE15 groups, respectively, compared to the BSRE0 group (*p* < 0.01). The fat content decreased linearly and quadratically in BSRE supplemented diets by 49, 53.03, and 63.23% for BSRE5, BSRE10, and BSRE15 groups, respectively, compared to the BSRE0 group (*p* < 0.01). The ash content was linearly and quadratically increased in the BSRE15 group by 37% compared to the BSRE0 group (*p* < 0.01).

**Figure 2:**
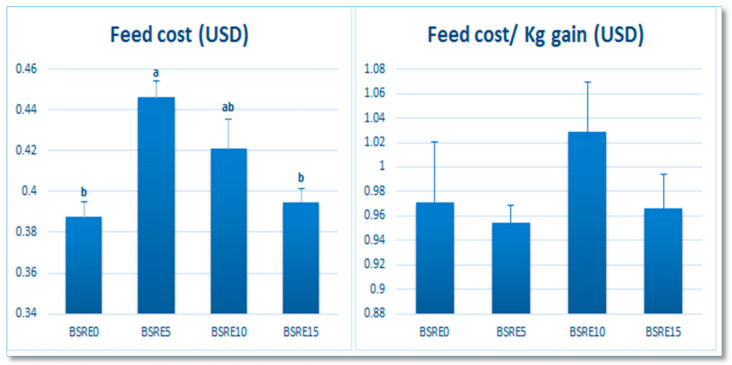
The effect of BSRE dietary addition on the economic value of the diets. Mean values in the same row with different superscripts differ significantly (*p* < 0.05). The data were expressed as the mean± standard error (SE). ^a, b^ Means within the same row carrying different superscripts are significantly different at (*p* < 0.05).

As shown in Figure 2, the feed cost was quadratically increased when fed on the BSRE5 diet compared to the BSRE0 diet and BSRE15 diet (*p* = 0.002). However, the feed cost/kg gain was not significantly different between all experimental diets (*p* > 0.05).

### 3.2. Serum Biochemical Parameters

As presented in Table 4, linear and quadratic reduction in the serum levels of glucose, total cholesterol, and triglycerides was detected in all BSRE supplemented treatments in a level-dependent manner. Dietary BSRE supplementation linearly and quadratically elevated the serum levels of total protein, albumin, α2 globulin, ɣ globulin, linearly raised the serum levels of total globulins, α1 globulin, and ß globulin in comparison with the control group (*p* < 0.05). The serum ALT level was linearly and quadratically decreased in the BSRE5 group and increased in the BSRE15 group compared to the BSRE0 group (*p* < 0.01). The serum level of creatinine was linearly reduced in the BSRE15 group compared to the BSRE0 group (*p* < 0.01).

### 3.3. Antioxidant Activity and Immune Indices

The effects of dietary BSRE on the antioxidant and immune status of *O. niloticus* are presented in Table 5. The linear and quadratic rise in the serum catalase activity and linear increase in the serum SOD activity and GSH level was observed in BSRE supplemented groups in a level-dependent manner (*p* < 0.01).

The serum lysozyme activity and MPO level were linearly and quadratically increased in BSRE supplemented groups in a level-dependent manner. The serum nitric oxide level was linearly and quadratically decreased in BSRE supplemented groups in a level-dependent way (*p* < 0.01).

### 3.4. Histological Finding

#### 3.4.1. Gills

**Figure 3:**
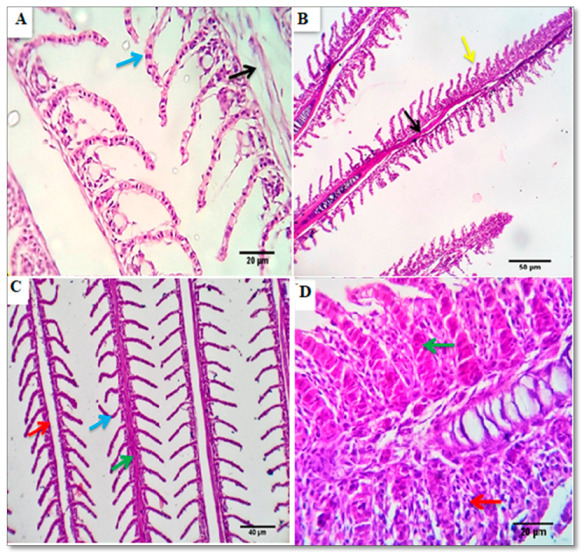
Photomicrograph from gills the control group (**A**) showing normal primary (black arrow) and secondary gill filaments with normal pavement cells, (blue arrow) Scale bars, 20 μm. (**B**) photomicrograph from the gills of the BSRE5 group, showing tips of a few filaments with epithelial lifting (yellow arrow) and stromal lymphocytic infiltration, Scale bars 50 μm. (**C**) photomicrographs from the gills of the BSRE10 group showing congested, Telangiectatic capillaries (green arrow), focal denudation of the secondary filament structures (blue arrow), lymphocytic infiltration (red arrow). Scale bars, 40 μm. (**D**) photomicrographs from the gills of the BSRE15 group showing that the tips of some filaments appear focally denuded, fused, thick, and enlarged by severely congested capillaries (green arrow), epithelial proliferation, goblet cell proliferation, and lymphocytic infiltration (red arrow).

Examined sections from the gills of the control group revealed normal histomorphological structures, the primary and secondary gill filaments were standard with normal pavement cells, lamellar epithelial cells, pillar cells, mucus-secreting cells (goblet cells), chloride cells, capillary channels (afferent and efferent venules), and a few mononuclear cells (Figure 3A). Gills of the BSRE5 group denoted minor changes, as the tips of a few filaments revealed epithelial lifting and stromal round cell infiltration (Figure 3B). Gills of the BSRE10 group revealed comparatively normal histomorphological structures in most of the examined cases; however, a few cases revealed focal changes at the tip of the gill filament represented by focal denudation of the secondary filament structures (epithelial lifting and necrosis, round cell infiltration and chloride cell proliferation (Figure 3C). Gills of the BSRE15 group pointed out comparatively somewhat morphopathological changes more prominent than the other groups. Some filaments’ tips appeared focally denuded, fused, thick, and enlarged by severely congested capillaries, epithelial proliferation, and round cell infiltration (Figure 3D).

#### 3.4.2. Intestine

**Figure 4:**
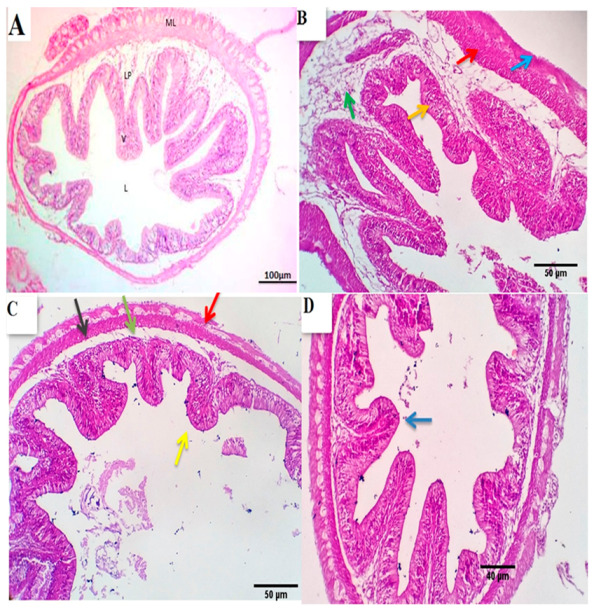
Photomicrograph from the posterior intestine of the control group (**A**) showing that the villi are broad and short, the crypts are deeper, the mucosal epithelial lining is free of cilia and contains more goblet cells. The mucosa shows a single epithelial layer of columnar cells, followed by the lamina propria and ill-distinct muscularis mucosa. The submucosa shows loose CT, reticular and elastic fibers beside some adipocytes. It encloses blood vessels and lymphatics. A thin mesothelial layer encoded the outermost muscle layer (serosa) Scale bars, 100 μm. (**B**–**D**) photo-micrographs from the posterior intestine of BSRE5, BSRE10, and BSRE15 groups shows nearly similar histomorphology as that of the control group, however, a mild to a moderate number of lymphocytes are seen infiltrating the lamina epithelialis, which is most prominent in the BSRE10 group (yellow arrows) Scale bars 50, 40 μm.

Investigated sections of the posterior intestine of the control group revealed a histomorphology similar to the anterior one; however, the villi are broad and short, the crypts are deeper, and the mucosal epithelial lining is free of cilia and contains more goblet cells. The mucosa appeared to be formed from a single epithelial layer of columnar cells, followed by the lamina propria and ill-distinct muscularis mucosa. The submucosa showed loose CT, reticular and elastic fibers beside some adipocytes. It encloses blood vessels and lymphatics. The muscular coat is formed from an inner circular and outer longitudinal smooth muscle fiber. A thin mesothelial layer encoded the outermost muscle layer (serosa) (Figure 4A). The posterior intestines of all BSRE treated groups were nearly similar to the control group; however, a mild to moderate number of lymphocyte infiltrating the lamina epithelialis was seen, which was most prominent in the BSRE10 group (Figure 4B–D). 

#### 3.4.3. Kidney

**Figure 5:**
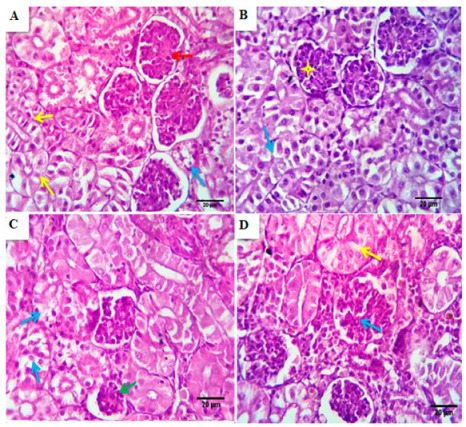
Photomicrographs from the kidney of the control group (**A**) showed normal renal glomerular, tubular, and interstitial structures with preserved Bowman’s capsular histomorphology, glomerular capillary morphology (red arrows), and tubular epithelial length and widths, minimal degenerative changes in a few numbers of renal tubules were seen (yellow arrows), Scale bar, 20 μm. (**B**–**D**) Photomicrographs from the kidney of BSRE5, BSRE10, and BSRE15 groups, respectively, showed comparatively healthy, normal counterparts of the nephron units with a slandered morphological appearance, which is homologous to the control one in BSRE5 group (blue arrows and yellow stars) and less standardized in BSRE10 and BSRE15 groups, where some of the renal tubules suffered degenerative changes, mostly of hydropic type ((**C**), blue arrows), ((**D**), yellow arrows), Scale bar, 20 μm.

Serial sections from the control group’s kidneys pointed out normal renal glomerular, tubular, and interstitial structures with preserved Bowman’s capsular histomorphology, glomerular capillary morphology, and tubular epithelial length and widths with a centrally located nucleus. Minimal degenerative changes were seen in a few numbers of renal tubules (Figure 5A). Kidneys of BSRE supplemented groups showed comparatively healthy, normal counterparts of the nephron units with a slandered morphological appearance which was more standardized and homologous to the control one in the BSRE5 and less standardized in BSRE10, BSRE15, where a more renal tubule suffered degenerative changes most of the hydropic type (Figure 5B–D).

#### 3.4.4. Spleen

**Figure 6:**
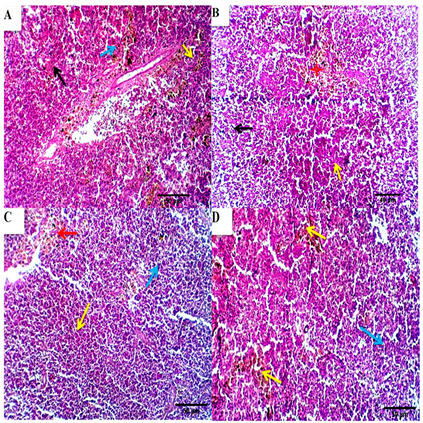
Photomicrographs from the spleen of the control group (**A**) showed characteristic proliferative aggregations of melano-macrophages, both perivascular and interstitial (blue arrow). The blood vessels and the splenic sinusoids were mild to moderately congested, and the latter occupied a large area of the tissue constituting the red pulp of the spleen (black arrow). Splenic cords are a mesh of fibroblast-like cells with foci of various blood cells. White pulp, consisting mainly of lymphoid cells, typically surrounds arterial vessels, assuming a nodular pattern (yellow arrows), scale bar; 50 μm. (**B**–**D**) photomicrographs from the spleen of BSRE5, BSRE10 and BSRE15 showed histomorphological structures comparable to the control group. The lymphoid elements appear more prevalent, particularly around the small size blood vessels forming aggregations or ill distinct nodular arrangement (yellow and blue arrows). The splenic cords were outstanding, especially in the BSRE10 group ((**C**), red arrows). Melano-macrophage centers (MMC) are mild to moderately activated ((**B**), red stars). Scale bars 50 μm.

The spleen of the control group showed characteristic proliferative aggregations of melano-macrophages, both perivascular and interstitial. The blood vessels and the splenic sinusoids were mild to moderately congested, and the latter occupied large areas of the tissue constituting the spleen’s red pulp. Splenic cords are a mesh of fibro-blast-like cells with foci of various blood cells. White pulp, consisting mainly of lymphoid cells, typically surrounds arterial vessels, sometimes assuming a nodular pattern. Melano macrophage centers (MMC) form small clusters in the parenchyma. The melano macrophage (MM) is a distinct immune cell type prevalent in the spleen (Figure 6A). The spleen of BSRE5, BSRE10, and BSRE15 revealed histomorphological structures comparable to the control group. Still, the lymphoid elements were more prevalent, particularly around the small size blood vessels forming aggregations or ill distinct nodular arrangement and the splenic cords were outstanding, especially in BSRE10. Melano-macrophage centers (MMC) were mild to moderately activated (Figure 6B–D).

### 3.5. Intestinal Morphometric Measurements

Table 6 highlights the morphometric measurements of the posterior section of the intestine. The villous height was linearly and quadratically increased in the BSRE5 group while it was decreased in BSRE10 and BSRE15 groups in comparison with the BSRE0 group (*p* < 0.01). The villous width was linearly and quadratically increased in BSRE supplemented group compared to the control group (*p* < 0.01). The mucosal thickness was linearly and quadratically increased in the BSRE5 and BSRE10 groups while it was decreased in the BSRE15 group (*p* < 0.01). Linear and quadratic increase in the intraepithelial lymphocyte infiltration was detected in the BSRE10 group and decreased in the BSRE5 group (*p* < 0.01). The goblet cell count was not significantly different between all experimental groups (*p* > 0.05).

### 3.6. Challenge Test

The dietary addition of BSRE improved the resistance of *O. niloticus* to *Staphylococcus aureus* challenge in terms of RPS % by 66.67, 75, 83.33% for BSRE5, BSRE10, and BSRE15 treatments, respectively.

## 4. Discussion

The data available for the effects of *Boswellia serrata* resin extract in fish diets are almost entirely scarce. Consequently, the current study was performed to illustrate the influence of BSRE as a feed additive on growth performance, general health, and immune response of *O. niloticus*. As an initial experiment, feeding *O. niloticus* on BSRE supplemented diets triggered no mortalities between all treated groups, which helped us eliminate the ID50. The present study reported improved FBW, TBWG, and total FI of fish fed on the BSRE5 diet. The PPV was increased in BSRE supplemented diets. However, no effect on the FCR by the addition of BSRE. The increased BW and BWG in the BSRE5 group in the current study may be due to the improved feed intake compared to the control group and other BSRE-complemented groups. As well, the improved growth performance in the BSRE5 diet may be attributed to the enhanced intestinal histology reported in this study that was indicated by increased villus height, villus width, and mucosal thickness, consequently improving the intestinal absorptive surface for nutrients. Amer et al. [8] reported that using phytogenic feed additives, particularly medicinal plants, leads to improved fish performance efficiency as it can augment gut function [9,10,48,49,50]. This coordinates with the intestinal morphometric measurements of the villus heights and the count of goblet cells, which secrete mucus to coat and keep the intestinal mucosa from damage, dehydration, and pathogens [51,52]. Caspary [53] determined that the intestinal villus length influences the absorption, which increases the feed utilization. Additionally, the higher intestinal villus heights and goblet cells count indicating an improvement in nutrient absorption, resistance against intestinal pathogens due to enhancing capacity of absorptive surface area, and so considered as a growth promoter. Gabriel et al. [54] recorded a marked improvement in the weight gain and specific growth rate in GIFT (genetically improved farmed tilapia) tilapia fed on aloe vera. Mukherjee et al. [55] stated enhanced growth and innate immunity of Nile tilapia by the dietary addition of *Withania somnifera* root extracts. However, higher levels of BSRE (10 and 15 g kg^−1^) did not affect the fish growth, which may be due to the hypolipidemic effect of BSRE bioactive compounds (boswellic acid and oleanolic acid), which observed in the fish body composition in the present study. Wang et al. [56] presented that oleanolic acid administration to mice (20 mg/kg/day) caused reduced body, fat, and liver weights.

Although the feed cost in the BSRE5 diet was increased, the feed cost/kg gain was not significantly different between all experimental diets. The increased feed cost was due to the increased feed intake in the BSRE5 group compared to other groups. However, due to the increased TBWG in the BSRE5 group compared to other groups, the feed cost/kg gain became not significantly different. Regarding the fish body composition results, dietary supplementation of BSRE increased the crude protein content and reduced the fat content in a level-dependent manner while the ash content was increased in the BSRE15 group. The obtained results may be attributed to the BSRE content from boswellic acid reported stimulating pancreatic enzyme secretion that improves protein and energy digestibility and decreases endogenous losses of nitrogen, ammonia production [57], in addition to having an antihyperlipidemic activity [58]. It was reported that oleanolic acid and boswellic significantly decreased visceral fat, plasma lipids, ghrelin, and increased leptin in obese Swiss mice [59].

Serum biochemical and innate immune parameters are crucial health pointers [60,61]. The blood glucose level is a significant physiological indicator estimating fish health conditions. It is mobilized to provide metabolic energy in fish [62] and used by fish to deal with physiological stress, acting as a useful stress tolerance indicator [62]. The BSRE acts as a hypoglycemic agent regarding serum glucose levels, which is a good sign of lowered stress [63]. The hypoglycemic effect of BSRE is due to its content from boswellic acid and oleanolic acid. The mechanism by which the boswellic acid induces the hypoglycemic effect is through increased peripheral glucose utilization and inhibition of intestinal glucose transporter activity, as reported in [58]. Whereas the hypoglycemic activity of oleanolic acid is carried out by improving the insulin response, it maintains the function and survival of β-cells and prevents complications of diabetes. Oleanolic acid may control the enzymes involved in insulin biosynthesis, signaling, and secretion [64]. Wang et al. [56] demonstrated that oleanolic acid’s hypoglycemic activity occurs through glucose improvement, insulin tolerance, enhancement of insulin signaling, and inhibition of gluconeogenesis.

Moreover, our results indicated a hypolipidemic effect of BSRE in a level-dependent manner, which could also be because of its content from boswellic acid and oleanolic acid. Jadhav and Puchchakayala [58] investigated the antihyperlipidemic activity of boswellic acid. Wang et al. [56] stated that oleanolic acid administration to mice (20 mg/kg/day) reduced the serum total cholesterol, triglyceride, LDL, and free fatty acids and decreased the accretion of hepatic lipid through downregulation of lipogenic genes expression (stearoyl-CoA desaturase 2 [SCD2], acetyl-CoA carboxylase [ACC], acyl-CoA cholesterol acyltransferase [ACAT], and glycerol-3-phosphate acyltransferase [Gpam]) [59]. Oleanolic acid and correlated triterpenes have important therapeutic properties such as antidiabetic, antioxidant, anti-inflammatory, microbicide, and hypolipidemic actions [65,66].

The current study showed a reduced ALT level in the BSRE5 group while its level was increased in the highest level of BSRE (BSRE15). Additionally, the creatinine level was reduced in the BSRE15 group. ALT is a standard indicator of liver disease, and increased serum levels indicate liver damage [67]. The reduced liver function also led to reduced creatine production and decreased creatinine levels in the blood due to reduced storage of creatine and reduced conversion of creatine to creatinine [68]. This indicates that increasing the level of BSRE badly affects liver function, which may be explained by liver overload by increasing the level of the bioactive compounds presents in BSRE that responsible for the stimulated immune system, hypoglycemic, and antihyperlipidemic effects where the liver is the site of production of serum total protein and its fractions and the organ of glucose and fat metabolism. The same was observed in the kidney and gill histoarchitectures, which showed moderate changes in BSRE10 and BSRE10 treatments.

Serum total protein, albumin, and globulin are effective indicators of humoral immunity and fish well-being as the essential components of blood serum, especially globulin, which is a protein fraction involved in the immune response [63,69], and its increase implies an enhancement in the fish immune system [10]. In the current study, the increased total serum protein, albumin, total globulin, and immunological globulin suggesting the immune-modulatory effect of BSRE in a level-dependent manner. Similarly, Syrovets et al. (2000) attributed the potent induction of the non-specific immune response by BSRE to their bioactive compounds (polysaccharides, incensol acetate, and boswillic acids), which act as an immune stimulant [12,70]. This supports our results of the increased intraepithelial lymphocytic infiltration and the splenic cords (Billroth’s cords or red pulp cords) outstanding, especially in BSRE10. These splenic cords are reservoirs of monocyte clusters that total more than the total number of monocytes in the circulation. They can be rapidly organized to leave the spleen and help treat persistent infections [71], indicating the improved ability to clear microbial antigens from the gut in BSRE10 and BSRE15, as the intestine is the primary infection route for pathogens in fish [72,73]. The melano-macrophage centers (MMC) were mild to moderately activated in splenic tissue of fish fed BSRE. El-Asely et al. [74] recorded increased MMC in Nile tilapia fed a diet supplemented by *Echinacea purpurea*. Additionally, Ledic-Neto et al. [75] stated that feeding Nile tilapia on a diet complemented with propolis (2%) for 15 days displayed increased melano-macrophage centers, despite the difference between treatments disappeared seven days later, suggesting physiological adaptation to the supplements.

Conversely, Brum et al. [76] found that at 55 days of feeding fish on a diet supplemented with 0.5% basil exhibited a reduction in the count of melano-macrophages centers. Despite this, there was no difference in the area of the spleen they engaged, and it could be said that the centers of macrophages and melanocytes in these animals were less distributed. Considering that their results were obtained after 55 days of feeding on a supplemented diet, it may be explained as a physiological adaptation to the supplemented diet. The current study results coordinate with Mukherjee et al. [55] who detected an elevation in the plasma levels of total protein and total immunoglobulin of Nile tilapia by dietary addition of *W. somnifera* root extracts.

The fish’s innate immunity is the main line of protection against invading pathogens. The lysozyme activity and nitric oxide (NO) are important components of the innate immune system that play a vital role in destroying pathogens [77]. Our results illustrated that BSRE increased the lysozyme activity in a level-dependent manner, which coordinates with the result of total protein and maybe explained by the potent non-specific stimulation of the innate immune system by BSRE bioactive compounds; polysaccharides, incensol acetate, and boswillic acids, which considered as an immune stimulant as mentioned by [12,70]. Engstad et al. [78] stated that increased lysozyme in the blood of stimulated fish is conducted either by the proliferated phagocytes or the increased productivity of lysosomes. Pratheepa and Sukumaran [79] demonstrated that the increased lysozyme activity could be because of increasing the blood neutrophils and monocytes of fish fed diet fortified with plant extract compared to that found in the control diet. The results of the present study are coordinated with Mukherjee et al. [55] who reported an increase in the lysozyme activity in Nile tilapia fed on a diet fortified with *W. somnifera* root extract. Furthermore, myeloperoxidase (MPO) is a neutrophil released enzyme that is an essential constituent of protection against pathogens [80] and has a vital function for the innate immune response [81]. The current study results showed increased MPO production in BSRE supplemented groups in a level-dependent manner that also indicates the immune-modulating effect of BSRE. These results harmonize with that obtained by Kurian et al. [81] who stated the activation of fish peroxidase when fed on herbal immune stimulants.

*Staphylococcus aureus* has been recently reported in Nile tilapia (*O. niloticus*) triggering high mortality with various pathological alterations [82]. It also poses health risks to fish handlers and consumers [83]. In addition, in aquaculture, methicillin-resistant *Staphylococcus aureus* (MRSA) has been isolated from tilapia in Malaysia [84]. It has also been related to mortality and morbidity in the culture of Nile tilapia in northern Egypt [85].

In the current study, BSRE supplementation increased the survivability of Nile tilapia challenged with *S. aureus* in a dose-dependent manner. The increased survivability may be due to the stimulation of innate and non-specific immune responses by BSRE supplementation and its bioactive compounds, which have antibacterial, immune-modulatory, anti-inflammatory properties [65,66,86]. Similarly, Kurian et al. [81] indicated that Nile tilapia fed Leucas Aspera showed markedly improved resistance against Streptococcus agalactiae infection compared to the control. The dietary addition of BSRE decreased the serum level of NO in a level-dependent manner. The reduced NO production can be attributed to the inhibition of the NO production and on the generation of NO synthase caused by the bioactive components of BSRE as sesquiterpenes [87], diterpenes [88,89], and triterpenes [90]. Furthermore, Yoshikawa et al. [91] indicated inhibition of nitric oxide production from lipopolysaccharide-activated macrophages caused by the mono and triterpenes constituents of *Boswellia carterii* gum-resin.

The antioxidant enzymes protect the fish body from oxidative alterations that resulted from increased ROS production [92,93]. The superoxide dismutase activity, glutathione peroxidase level, and catalase activity are significant indicators for estimating the antioxidant activity in aquatic organisms [94,95]. Our study reported increased levels of antioxidant enzymes (CAT, SOD, and GSH) in BSRE supplemented groups in a level-dependent manner that can be attributed to the bioactive triterpenes (boswellic acids and oleanolic acid) that possess antioxidant activity [96,97,98] through increasing the expression of the antioxidant enzymes [64]. Similarly, Sharma et al. [99] reported the antioxidant activity of aqueous extract of *B. serrate* in a level-dependent manner. Afsar et al. [100] demonstrated the invitro antioxidant activity of methanolic extract of *Boswellia serrate* due to its content of flavonoids, terpenoids, tannins, saponins, anthraquinones.

## 5. Conclusions

Dietary addition of *Boswellia serrata* resin extract can enhance the antioxidant activity, immune status, and disease resistance of *O. niloticus* to *S. aureus* infection in a level-dependent manner. The 5 g kg^−1^ BSRE level can improve fish growth without causing harmful effects on fish health. The highest levels of BSRE are not recommended as they badly affected the histoarchitecture of many vital organs. Further research is recommended to be carried out on lower levels of BSRE and to evaluate the effects of its addition on the histopathological examination of the liver.

## Figures and Tables

**Table 1:** Feed formulation and proximate composition (g kg^−1^ on a dry weight basis).

Ingredients	g kg^−1^
Soybean meal 49% CP	319.4
Fish meal 70.7% CP	150
Yellow corn	157.1
Corn gluten 67% CP	100
Wheat flour	100
Wheat bran	80
Fish oil	60
Methionine	3.5
Premix ^1^	30
Chemical composition (g kg^−1^)	
Crude protein	374.76
Crude fiber	40.59
Fat	94.74
NFE ^2^	427.53
Ash	62.35
Lysine	20.321
Methionine	10.88
GE MJ/kg ^3^	20.70

^1^ Composition of vitamins and minerals premix kg^−1^: vitamin A580,000 IU; vitamin D3 8600 IU; vitamin K3 142 mg; vitamin E 720 mg; vitamin C 0.1 mg; vitamin B1 58 mg; vitamin B2 34 mg; vitamin B6 34 mg; vitamin B12 58 mg; folic acid 86 mg; biotin 50 mg; pantothenic acid 8 mg; zinc methionine 3000 mg; manganese sulfate 65 mg; copper sulfate 3400 mg; iron sulfate 2000 mg; sodium selenite 25 mg; cobalt sulfate 572 mg; calcium iodide 25 mg; calcium carbonate as carrier up to till 1 kg. ^2^ Nitrogen free extract, determined by difference = 100 − (protein % + fat % + crude fiber % + ash %). ^3^ Gross energy (GE) was calculated according to NRC (2011) as 23.6 KJ/g protein, 39.5 KJ/g lipid and 17.0 KJ/g NFE.

**Table 2:** The effects of dietary *Boswellia serrate* resin extract (BSRE) on the growth performance of *O. niloticus*.

Parameters	BSRE0	BSRE5	BSRE10	BSRE15	SEM	Regression Analysis #	
						Linear	Quadratic
IBW/fish (g)	22.38	21.71	22.32	20.85	0.48	0.72	0.49
FBW/fish (g)	55.72 ^b^	60.71 ^a^	56.45 ^b^	54.96 ^b^	0.76	0.12	0.005
TBWG/fish(g)	33.34 ^b^	39.00 ^a^	34.13 ^b^	34.10 ^b^	0.78	0.54	0.01
ADFI/ fish (g)	0.74 ^b^	0.85 ^a^	0.80 ^ab^	0.75 ^b^	0.01	0.94	0.002
Total FI/fish (g)	41.68 ^b^	48.00 ^a^	45.28 ^ab^	42.47 ^b^	0.87	0.94	0.002
FCR	1.25	1.23	1.32	1.24	0.02	0.73	0.54
PER	2.13	2.16	2.01	2.14	0.03	0.69	0.52
PPE	0.98 ^b^	1.43 ^a^	1.28 ^a^	1.33 ^a^	0.05	0.001	0.001

# The regressions were considered significant at *p* < 0.05. IBW, Initial body weight; FBW, Final body weight; TBWG, Total bodyweight gain; FI, feed intake; FCR, Feed conversion ratio; PER, Protein efficiency ratio; PPE, Protein productive effi-ciency. Variation in the data was expressed as pooled SEM; Mean values in the same row with different superscripts differ significantly (*p* < 0.05). ^a, b^ Means within the same row carrying different superscripts are significantly different at (*p* < 0.05).

**Table 3:** The effects of dietary *Boswellia serrate* resin extract (BSRE) on the proximate whole-fish body composition of *O. niloticus.*

Parameters	Initial	BSRE0	BSRE5	BSRE10	BSRE15	SEM	Regression Analysis #	
							Linear	Quadratic
DM% *	20.55	24.13	27.45	25.05	27.17	0.57	0.06	0.37
Crude protein % **	54.83	45.58 ^d^	65.92 ^a^	63.68 ^b^	61.86 ^c^	2.42	0.00	0.00
Crude lipids % **	18.64	36.39 ^a^	18.50 ^b^	17.09 ^b^	13.38 ^c^	2.69	0.00	0.00
Ash% **	22.36	15.35 ^bc^	13.89 ^c^	16.96 ^b^	21.03 ^a^	0.83	0.00	0.001

# The regressions were considered significant at *p* < 0.05. Variation in the data was expressed as pooled SEM; Mean values in the same row with different superscripts differ significantly (*p* < 0.05). * On fresh basis, ** on dry matter basis. ^a,b,c,d^ Means within the same row carrying different superscripts are significantly different at (*p* < 0.05).

**Table 4:** The effects of dietary *Boswellia serrate* resin extract (BSRE) on the blood biochemical parameters of *O. niloticus.*

Parameters	BSRE0	BSRE5	BSRE10	BSRE15	SEM	Regression Analysis #	
						Linear	Quadratic
Triglycerides (mg dL^−1^)	324.50 ^a^	255.00 ^b^	209.00 ^c^	160.50 ^d^	18.22	0.00	0.00
Cholesterol (mg dL^−1^)	220.50 ^a^	213.66 ^b^	206.50 ^c^	168.50 ^d^	6.07	0.00	0.00
Glucose (mg dL^−1^)	71.00 ^a^	68.30 ^b^	62.65 ^c^	61.65 ^c^	1.19	0.01	0.00
Total protein (g dL^−1^)	6.10 ^d^	7.70 ^c^	8.35 ^b^	8.90 ^a^	0.32	0.00	0.001
Albumin (g dL^−1^)	2.00 ^b^	2.35 ^ab^	2.40 ^ab^	2.55 ^a^	0.07	0.008	0.02
Total globulin (g dL^−1^)	4.90 ^c^	5.35 ^bc^	5.80 ^b^	6.50 ^a^	0.18	0.00	0.40
α1 globulin (g dL^−1^)	1.10 ^b^	1.27 ^a^	1.32 ^a^	1.35 ^a^	0.03	0.001	0.06
α2 globulin (g dL^−1^)	1.30 ^d^	1.52 ^c^	1.67 ^b^	1.78 ^a^	0.05	0.00	0.003
ß globulin (g dL^−1^)	1.10 ^c^	1.12 ^bc^	1.30 ^ab^	1.35 ^a^	0.03	0.002	0.78
ɣ globulin (g dL^−1^)	0.95 ^c^	1.40 ^b^	1.90 ^a^	2.10 ^a^	0.13	0.00	0.04
ALT	23.00 ^b^	18.70 ^c^	21.65 ^b^	31.45 ^a^	0.01	0.00	0.00
Creatinine	0.11 ^a^	0.10 ^a^	0.10 ^a^	0.07 ^b^	0.005	0.00	0.10

# The regressions were considered significant at *p* < 0.05. Variation in the data was expressed as pooled SEM; Mean values in the same row with different superscripts differ significantly (*p* < 0.05). ^a,b,c,d^ Means within the same row carrying different superscripts are significantly different at (*p* < 0.05).

**Table 5:** The effects of dietary *Boswellia serrate* resin extract (BSRE) on the antioxidant and immune status of *O. niloticus.*

Parameters	BSRE0	BSRE5	BSRE10	BSRE15	SEM	Regression Analysis #	
						Linear	Quadratic
Antioxidant capacity							
SOD (U L^−1^)	3.77 ^d^	4.77 ^c^	6.36 ^b^	7.08 ^a^	0.39	0.00	0.39
CAT (U L^−1^)	124.00 ^d^	162.50 ^c^	190.00 ^b^	201.50 ^a^	9.01	0.00	0.00
GSH (mmol L^−1^)	2.67 ^b^	3.05 ^b^	3.75 ^a^	3.78 ^a^	0.14	0.00	0.11
Immunological indices							
Lysozyme (µg mL^−1^)	4.61 ^c^	5.23 ^b^	6.42 ^a^	6.41 ^a^	0.23	0.00	0.00
MPO (U L^−1^)	18.50 ^d^	26.40 ^c^	30.10 ^b^	36.65 ^a^	1.99	0.00	0.00
NO (µmol L^−1^)	79.50 ^a^	40.50 ^b^	31.50 ^c^	29.00 ^d^	6.12	0.00	0.00

# The regressions were considered significant at *p* < 0.05. Variation in the data was expressed as pooled SEM; Mean values in the same row with different superscripts differ significantly (*p* < 0.05). CAT: catalase, SOD: superoxide dismutase, GSH: reduced glutathione, NO: nitric oxide, MPO: myeloperoxidase. ^a,b,c,d^ Means within the same row carrying different superscripts are significantly different at (*p* < 0.05).

**Table 6:** The effects of dietary *Boswellia serrate* resin extract (BSRE) on the morphometric measures of the intestine of *O*. *niloticus*.

Parameters	BSRE0	BSRE5	BSRE10	BSRE15	SEM	Regression Analysis #	
						Linear	Quadratic
Villous height (µm)	583.94 ^b^	709.31 ^a^	546.61 ^c^	418.88 ^d^	31.44	0.00	0.00
Villous width (µm)	208.25 ^d^	212.46 ^c^	232.32 ^b^	390.47 ^a^	22.71	0.00	0.00
Mucosal thickness (µm)	143.27 ^c^	266.63 ^a^	162.57 ^b^	140.09 ^d^	15.60	0.00	0.00
Goblet cell count	2.33	3.66	1.66	2.66	0.26	0.52	0.63
IELI	34.00 ^b^	17.00 ^c^	79.00 ^a^	37.33 ^b^	6.88	0.00	0.00

# The regressions were considered significant at *p* < 0.05. Variation in the data was expressed as pooled SEM; Mean values in the same row with different superscripts differ significantly (*p* < 0.05). IELI: intraepithelial lymphocyte infiltration. ^a,b,c,d^ Means within the same row carrying different superscripts are significantly different at (*p* < 0.05).

## Data Availability

Data sharing not applicable.
